# Vision-related quality of life in first stroke patients with homonymous visual field defects

**DOI:** 10.1186/1477-7525-8-33

**Published:** 2010-03-26

**Authors:** Carolin Gall, Gabriele H Franke, Bernhard A Sabel

**Affiliations:** 1 Otto-von-Guericke University of Magdeburg, Medical Faculty, Institute of Medical Psychology, Leipziger Str. 44, 39120 Magdeburg, Germany; 2 University of Applied Sciences Magdeburg-Stendal, AHW, Department of Rehabilitation Psychology, Stendal, Germany

## Abstract

**Background:**

To evaluate vision-related and health-related quality of life (VRQoL, HRQoL) in first stroke patients with homonymous visual field defects (VFD) with respect to the extent of the lesion. Since VFD occur in approximately 10% of stroke patients the main purpose of the study was to investigate the additional impact of VFD in stroke patients hypothesizing that VFD causes diminished VRQoL.

**Methods:**

In 177 first stroke patients with persisting VFD 2.5 years after posterior-parietal lesions VRQoL was assessed by the National-Eye-Institute-Visual-Functioning-Questionnaire (NEI-VFQ) and HRQoL by the Medical-Outcome-Study Short-Form-36 Health-Survey (SF-36). Questionnaire results of VFD-patients were compared with age- and sex-matched healthy controls and with general non-selected stroke samples as published elsewhere. VFD-type and visual acuity were partially correlated with questionnaire results.

**Results:**

Compared to healthy controls VFD-patients had lower NEI-VFQ scores except ocular pain (Z-range -11.34 to -3.35) and lower SF-36 scores except emotional role limitations (Z-range -7.21 to -3.34). VFD-patients were less impaired in SF-36 scores than general stroke patients one month post lesion (6/8 subscales) but had lower SF-36 scores compared to stroke patients six months post lesion (5/8 subscales). Visual acuity significantly correlated with NEI-VFQ scores (r-range 0.27 to 0.48) and VFD-type with SF-36 mental subscales (r-range -0.26 to -0.36).

**Conclusions:**

VFD-patients showed substantial reductions of VRQoL and HRQoL compared to healthy normals, but better HRQoL compared to stroke patients one month post lesion. VFD-patients (although their lesion age was four times higher) had significantly lower HRQoL than a general stroke population at six months post-stroke. This indicates that the stroke-related subjective level of HRQoL impairment is significantly exacerbated by VFD. While VRQoL was primarily influenced by visual acuity, mental components of HRQoL were influenced by VFD-type with larger VFD being associated with more distress.

## Background

Homonymous visual field defects (VFD) are among the most common disorders after posterior-parietal strokes and can severely reduce vision-related quality of life (VRQoL) [1-3]. It is known that diminished VRQoL is correlated with the extent of visual field loss after cerebral injury [1-3]. A correlation between visual field loss and quality of life was also shown in a large population-based cross-sectional study [4] and for different ophthalmologic diseases resulting in VFD such as glaucoma [5-11], retinal lesions [12,13] or optic neuropathy [14] (An overview of these studies which investigated the association of visual field impairment and quality of life is given in an additional file 1).

The impact of VFD on health-related quality of life (HRQoL) in general and VRQoL in particular, assessed in first stroke patients with VFD, has not yet been investigated in sufficient detail. Two studies with small sample sizes showed that diminished vision-related QoL is moderately correlated with the extent of visual field loss after cerebral injury to the postchiasmatic pathway. While one study focused on the area of sparing within the affected half of the visual field [1], the second study took the total area of visual field loss as the relevant parameter [2]. However, the etiology of these studies was not restricted on first stroke. In a recent study on VRQoL and HRQoL, we investigated a large sample of 312 brain-injured patients with postchiasmatic VFD and observed a coordinate influence of VFD and visual acuity on VRQoL in particular, but also on HRQoL [3]. The etiology of this sample was quite heterogenous and did not allow us to conclude on quality of life in first stroke patients with VFD.

There are several studies focussing on HRQoL among stroke patients during the course of rehabilitation or on long-term follow-up [15-18]. HRQoL assessments are an essential evaluation tool in healthcare and medical treatments [19], but usually measures such as neurological scores and disability scales are used. These are of only limited value to capture changes of the patient's subjective health status and insensitive to assess if patients have fully regained independence in everyday life [20]. The latter is the case in two thirds of the stroke patients who are alive 6 months after the lesion [21]. The most frequently used disability scales are the Barthel ADL Index [22] and the Functional Independence Measure [23] both commonly used to show improvements in functional status during inpatient stroke rehabilitation. However, because of ceiling effects these kinds of measures do not capture deficits in more advanced activities in the visual domain such as 'going down steps, stairs, or curbs in dim light or at night', 'seeing how people react to things you say' or 'driving at night'. These examples are items included in the National-Eye-Institute-Visual-Functioning-Questionnaire (NEI-VFQ) which is an appropriate measure for VRQoL. Stroke patients with VFD after older lesions but persistent vision problems often adapt to or compensate for their deficit and achieve functional independence, resulting in relatively normal Barthel scores. Nevertheless, these patients still have deficits in more advanced visual activities resulting in considerably diminished VRQoL [3].

One aim of the present study was to assess VRQoL and HRQoL in first stroke VFD-patients and to compare the results with those of age- and sex-matched healthy controls. Differences in self-rated VRQoL of more than 10 points are considered as clinically relevant [24,25]. The main purpose of the study was to investigate the additional impact of visual field loss in stroke patients on quality of life estimates hypothesizing that quality of life - especially VRQoL - is lower in stroke patients with than in stroke patients without VFD. Since HRQoL of first stroke VFD-patients has not yet been contrasted with general stroke patients with non-selected etiologies the primary aim of the present study was to capture this comparison. Both VRQoL and HRQoL estimates of VFD-stroke patients were further correlated with demographic and lesion variables, VFD-type and visual acuity. In addition, the influence of VFD size and visual acuity on VRQoL and HRQoL were investigated by analyses of variance.

## Methods

### Subjects

All analyses were based on data concurrently collected in two independent outpatient facilities for neurovisual rehabilitation (Institute of Medical Psychology and NovaVision center of excellence for visual therapy) in Magdeburg, Germany, between 1998 and 2007 [3]. Patients who met the following criteria were included in the study: (1) first posterior-parietal stroke; (2) clinical evidence of VFD in computer based perimetry; (3) willingness to participate in visual field diagnostics and questionnaire assessment, able to make the required study visits, and sufficient ability to follow instructions; (4) age 18 or older, with no upper age limit; (5) lesion older than 6 months; (6) absence of recurrent stroke according to medical records.

Exclusion criteria were severe psychotic diseases, serious drug abuse, chronic degenerative diseases (dementia, multiple sclerosis), severe motor impairments (paresis in both arms), noticeably low intelligence, considerably impaired visual acuity (corrected decimal binocular acuity < 0.4 respectively > 0.4 LogMAR acuity) or inability to fixate. First stroke patients with VFD associated with hemispatial neglect were excluded from the analyses (35) as well as patients with brain injuries with etiologies different from first stroke, i.e. recurrent stroke (25), non-progressive or extirpated brain tumors (38), traumatic brain injury (30), encephalitis (4), ectomy for epilepsy (2), and anoxic brain (1).

All patients were treated according to the ethical standards of the Declaration of Helsinki (1964). Ethical approval was not obtained according to local regulations because the present study required only answering questions without risk of psychological distress. For self assessment NEI-VFQ-39 and SF-36 questionnaires were sent to the patients by mail [26]. All patients were informed that answering the questionnaires was voluntary. Patients were asked to answer the questionnaires without help. All included subjects were able to comprehend the questions contained in the NEI-VFQ and SF-36.

Out of a total sample of 312 patients with cerebral injury resulting in postchiasmatic VFD 177 first stroke patients were selected for data analyses. Lesions were either ischemic (139) or hemorrhagic (38). Mean age was 57.4 years (SD = 13.76, range 21-83). 114 patients (64.4%) were male, 63 (35.6%) female. Mean lesion age was 30.69 (months) (SD = 40.30, range 6-277), i.e. on average more than 2.5 years. The type of VFD was complete hemianopia (n = 34), incomplete hemianopia (n = 72), quadrantanopia (n = 31), tunnel vision (n = 5), scotoma (n = 3), diffuse loss of vision (n = 23) and VFD affecting three quadrants (n = 9).

The following data were collected in this sample: NEI-VFQ (VRQoL) and SF-36 (HRQoL), demographic data, stroke-type (i.e. ischemic or hemorrhagic), visual field examinations, topography of the visual field loss (i.e. VFD-type), and visual acuity.

### Vision-related quality of life

The NEI-VFQ was originally designed to measure VRQoL in patients with chronic eye diseases [27]. In the present study the validated German 39-item version of the NEI-VFQ was used in self-administered format [28]. The questionnaire consists of 39 rating items with 12 subscales: (1) general health (2 items); (2) general vision (2 items); (3) ocular pain (2 items); (4) difficulties with near vision activities (6 items); (5) difficulties with distance vision activities (6 items); (6) limitations in social functioning due to vision (3 items); (7) mental health symptoms due to vision problems (5 items); (8) role difficulties due to vision problems (4 items); (9) dependency on others due to vision problems (4 items); (10) driving problems (3 items); (11) color vision problems (1 item) and (12) peripheral vision problems (1 item). A composite score was generated by averaging the 11 vision-related subscales without general health. Subscale and composite scores ranged from 0 ("worst possible functioning") to 100 ("best possible functioning"). NEI-VFQ reference values of a German sample of healthy control subjects were used for comparison [29].

### Health-related quality of life

The Medical Outcome Study Short-Form 36 Health Survey (SF-36) is a standard instrument for the assessment of general HRQoL. This questionnaire was used to quantify HRQoL in patients, independent of their actual state of health or their age. The questionnaire consists of 36 items subdivided into eight dimensions of subjective health: physical functioning (10 items), role limitations due to physical problems (4 items), bodily pain (2 items), general health perceptions (5 items), vitality (4 items), social functioning (2 items), role limitations due to emotional problems (3 items), and emotional well-being (5 items). All items can be combined to form two summary scales: the physical composite score and the mental composite score. Composite scores were generated by adding the item responses and including given loadings for the different dimensions. Subscale and composite scores ranged from 0 ("worst possible functioning") to 100 ("best possible functioning"). In the present study the German translation of the SF-36 was self-administered and patients were asked to rate the items based on the experiences during the last four weeks [30]. For comparison, SF-36 reference data of a German sample of healthy control subjects were derived from Bullinger & Kirchberger [30]. The reference sample also answered the SF-36 considering the time frame of the last four weeks.

### Visual field diagnostics

The VFD-type was assessed as tunnel vision, VFD affecting three quadrants, complete hemianopia, incomplete hemianopia, quadrantanopia, scotoma or diffuse loss of vision. The diagnosis of the defect type was based on campimetric (16° vertically × 21.5° horizontally, "High Resolution Perimetry, HRP") and perimetric 90° visual field measurements [31]. During a campimetric test 474 light stimuli were presented in a dense grid of 19 × 25 stimulus locations. At least 70 times during a campimetric visual field test, fixation accuracy was tested by an isoluminant change of the fixation point.

The campimetric visual field test was repeated three times. The mean number of correctly detected stimuli in campimetry in % served as an estimate for intact central visual field and was 57.83% (SD = 16.56). Reliability of the campimetric visual field examination was sufficient: the percentage of false positive responses was 2.32% (SD = 4.79), mean fixation accuracy was 93.09% (SD = 11.82%).

The eccentricity of the VFD was analyzed in a subsample of 90 patients with available digital visual field data. This subsample did not differ from the remaining 87 patients with respect to the mean number of correctly detected stimuli and reliability parameters. At each of the 474 tested positions three stimuli were presented, i.e. one during each test. Since campimetry was performed three times, a patient could detect between 0 and 3 out of 3 presented stimuli resulting in detection rates between 0 and 1. The detection rate at each tested position was multiplied by the eccentricity of the respective position. These 474 detection rates weighted by eccentricity were added and divided by 474 resulting in an individual value representing the mean eccentricity of intact visual field.

### Visual acuity

Best corrected visual acuity and reading speed were measured at a 0.4 m distance with Landolt, Snellen or the German-language Radner Reading Charts [32]. Visual acuity scores were analyzed through the calculation of weighted average LogMAR (WMAR) [33,34]. The numerator of the visual acuity score was divided by the denominator, and the base 10 logarithm of the result was calculated. WMAR then summarized the acuity data from both eyes in one score giving a 0.75 weighting to the better eye and a 0.25 weighting to the worse eye. Visual acuity scores were finally percentage transformed.

### Statistical analyses

NEI-VFQ and SF-36 scores of the first stroke sample were compared to reference values of age- and sex-matched healthy controls with the Wilcoxon test. The NEI-VFQ reference group (mean age = 49.88; SD = 16.8; range 21-79) consisted of 353 healthy controls (54.7% female) that was recently analyzed as a control group for stroke patients with homonymous visual field loss [29]. There were no differences concerning age and sex between the present first stroke sample and the healthy controls.

SF-36 reference data was derived from values published in the German SF-36 manual [30] of a control group consisting of 2914 healthy controls (age range 14 to >70 years, only persons older than 21 years were chosen for the present study).

The total sample of first stroke patients with VFD was subdivided into six age categories separately for males and females (21-30, 31-40, 41-50, 51-60, 61-70, >70 years). Mean NEI-VFQ and SF-36 scores of the corresponding sex and age-category were assigned to each VFD-patient. Thus, the group comparison was performed with averaged reference values specific to the first stroke sample. There were no differences concerning age and sex between the present first stroke sample and the healthy controls.

Standard-Deviation-Scores (SDS) were calculated as average NEI-VFQ respectively SF-36 subscale scores in the first stroke sample minus corresponding average values of healthy controls divided by the standard deviation of healthy controls [29,30]. SDS-scores were also evaluated for patients with different lesion ages (1 and 6 months), previously published by Rønning and Stavem [17]. 179 stroke patients aged ≥ 60 years with intracerebral haemorrhage and prior stroke(s) were included in this study [17]. Since Rønning and Stavem did not report values for SF-36 physical and mental composite scores, reference values reported by Suenkeler et al. [35] for both composite scores were used for evaluating SDS-scores. The authors studied HRQoL in 144 ischemic or hemorrhagic stroke/TIA patients (mean age 65.3 years) at 3, 6 and 12 months post stroke [35].

Partial parametric correlation coefficients were calculated between NEI-VFQ and SF-36 composite and subscale scores and age, lesion age, visual acuity and computer campimetry results. For nonparametric variables (sex, etiology, type of VFD) partial gamma correlations were calculated.

For further analyses the sample was divided into four groups according to their residual intact central visual field, measured as the number of correctly detected stimuli in campimetry (in %): 0-25%, 26-50%, 51-75% and 76-100%. Group differences were also studied for the factor visual acuity. Therefore, patients were assigned to one of the two groups: 0-50% and >50% visual acuity (0% corresponds to 0.4 decimal acuity respectively 0.4 LogMAR acuity). Mean NEI-VFQ and SF-36 composite and subscale scores were compared between groups with different intact visual field size and with different levels of visual acuity using analyses of variance with post-hoc t-tests in case of significant main effects. The level of significance was adjusted by the number of subscale comparisons (NEI-VFQ: 0.05/12 = 0.00417; SF-36: 0.05/8 = 0.00625).

Results were displayed as mean ± standard deviation (*M *± *SD*) concerning averaged questionnaire results and as mean ± standard error (*M *± *SE*) in case of SDS-scores. Statistical analyses were carried out with SPSS 15.0.

## Results

### Comparison of quality of life estimates between healthy controls and patients with VFD

Compared with healthy age- and sex-matched control subjects first stroke VFD-patients had significantly lower VRQoL in the NEI-VFQ composite score and in 11 of 12 NEI-VFQ subscales, Wilcoxon *Z*-range -3.35 to-11.34; all *P *< 0.001, (Table 1). Only the subscale ocular pain did not differ to healthy controls (*Z *= -1.34; *n.s*). Between group differences exceeded more than 10 points for 10/12 subscales; the subjective impairment was therefore considered as clinically relevant [24,25].

**Table 1:** NEI-VFQ and SF-36 results of first stroke patients with VFD compared with healthy age- and sex-matched controls

	First Stroke Patients		Healthy Controls^1,2^		Mean difference between samples	Z^1 ^(*P*)
	
	M	SD	M	SD		
NEI-VFQ (N)						

NEI-VFQ composite score (177)	63.98	16.89	92.06	4.73	-28.08	-10.54 ‡
1. General health (173)	49.14	19.9	63.93	11.89	-14.79	-7.04 ‡
2. General vision (173)	57.23	17.88	78.72	8.39	-21.49	-10.48 ‡
3. Ocular pain (175)	86.86	16.04	86.19	7.21	0.67	1.34 (n.s.)
4. Near vision (177)	65.25	22.69	89.17	9.38	-23.92	-10.17 ‡
5. Distance vision (177)	72.75	21.31	91.12	8.94	-18.37	-8.82 ‡
6. Social functioning (177)	74.65	23.33	93.62	7.77	-18.97	-8.83 ‡
7. Mental health (174)	59.43	24.19	86.17	11.65	-27.28	-9.64 ‡
8. Role difficulties (175)	51.87	22.59	90.01	5.26	-38.14	-11.34 ‡
9. Dependency (173)	67.21	30.47	93.78	7.51	-26.57	-8.61 ‡
10. Driving (153)	27.35	33.89	88.30	8.02	-60.95	-9.43 ‡
11. Color vision (172)	86.92	22.69	94.26	6.54	-7.34	-3.35 ‡
12. Peripheral vision (175)	49.29	24.19	92.06	8.21	-42.77	-11.03 ‡

SF-36 (N)						

1. Physical functioning (173)	66.41	27.10	80.53	10.06	-14.12	-4.96 ‡
2. Role limitations (physical) (174)	47.99	43.30	78.82	8.79	-30.81	-7.21 ‡
3. Bodily pain (174)	81.06	24.76	74.79	6.42	6.27	3.41 ‡
4. General health perceptions (173)	56.37	21.13	62.61	6.02	-6.24	-3.34 ‡
5. Vitality (176)	53.25	19.88	62.03	3.65	-8.78	-5.14 ‡
6. Social functioning (176)	74.79	26.34	87.56	2.66	-12.77	-5.01 ‡
7. Role limitations (emotional) (170)	71.76	42.75	89.21	2.78	-17.45	-1.29 (n.s.)
8. Emotional well-being (176)	66.64	18.91	74.67	2.71	-8.03	-4.54 ‡

* *P *< 0.05; † *P *< 0.01; ‡ *P *< 0.001; ^1 ^NEI-VFQ reference values [29]. SF-36 reference values [30]. α-adjusted significance-level is 0.00417 for NEI-VFQ and 0.00625 for SF-36. Healthy controls were matched by sex and age.

Comparison of first stroke VFD-patients with healthy SF-36 control values from Bullinger & Kirchberger [30] revealed lower HRQoL scores in VFD-patients in 7 of 8 SF-36 scales, Wilcoxon *Z*-range: -3.34 to-7.21; all *P *< 0.001, (Table 1). The difference between the samples for role limitations due to emotional problems did not reach significance. VFD-patients had higher scores than controls in the subscale bodily pain (*Z *= 3.41; *P *< 0.01).

**Figure 1:**
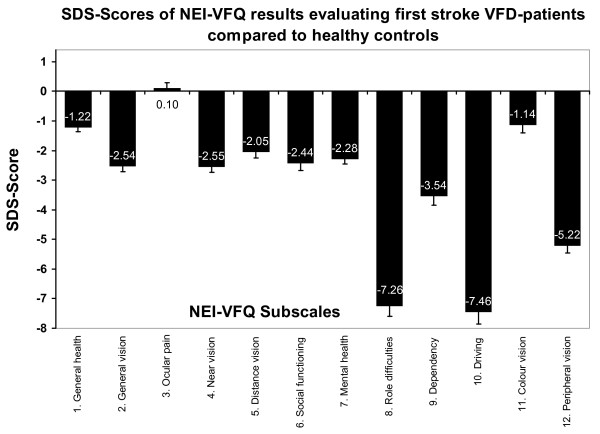
**SDS-scores for NEI-VFQ of first stroke VFD-patients compared with a healthy reference group**. SDS was calculated as average NEI-VFQ subscale scores in the first stroke VFD-sample minus the average value of healthy NEI-VFQ control subjects divided by the standard deviation of the control sample. The zero-line represents the baseline value of the control group sample without stroke. All NEI-VFQ SDS-scores (except ocular pain) are negative indicating that first stroke VFD-patients suffer from lower VRQoL than healthy controls.

Figure 1 demonstrates the relation between diminished VRQoL of first stroke VFD-patients relative to healthy controls with the aid of SDS-scores. Except for the subscale ocular pain, NEI-VFQ results of first stroke VFD-patients were always below average scores of age- and sex-matched controls (Figure 1). The mean NEI-VFQ SDS-score was -3.36 (SD = 2.13). Role difficulties, driving and peripheral vision showed the largest deviations with SDS-scores below -5.

**Figure 2:**
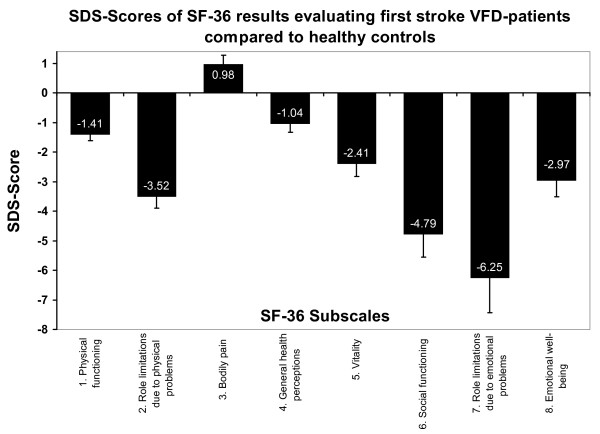
**SDS-scores for SF-36 results of first stroke VFD-patients compared to a healthy reference group**. Data of healthy reference subjects [30]. Only the SDS-score for the subscale bodily pain was positive which indicates that first stroke VFD-patients suffer from lower HRQoL than healthy controls.

Relating SF-36 values of VFD-patients to healthy controls SDS-scores for all scales except for bodily pain were below the average of healthy controls. Only the SDS of role limitations due to emotional problems deviated by more than -5 (Figure 2). Mean SF-36 SDS-score was -2.66 (SD = 5.07).

### Comparison of quality of life estimates between general stroke samples and patients with VFD

**Figure 3:**
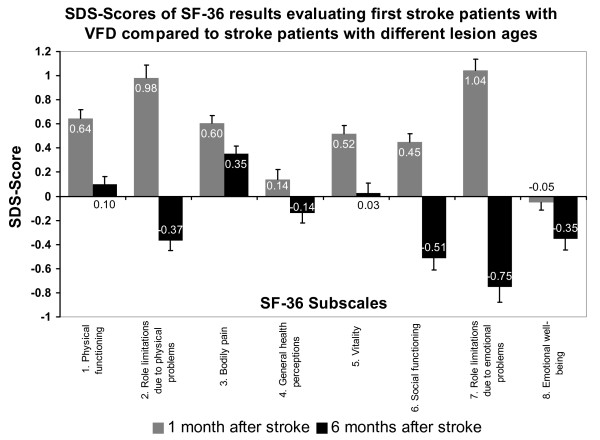
**SDS-scores for SF-36 subscales of first stroke VFD-patients compared to stroke patients with different lesion ages**. Data of stroke patients with different lesion ages [17]. SDS was calculated as average SF-36 subscale score in the first stroke VFD-sample minus average value of stroke patients one months post lesion (grey) or six months post lesion (black) divided by the standard deviation of the stroke groups with different lesion ages.

Figure 3 shows SDS-scores comparing the sample of first stroke patients with VFD with stroke patients in general. The SF-36 results of these stroke patients with different lesion ages (1 month vs. 6 months) were originally published by Rønning and Stavem [17]. VFD-patients showed significantly better SF-36 scores than stroke patients with a lesion age of 1 month (Z-range -6.56 to -9.29; *P *< 0.001) except for the subscales general health perceptions (Z = -1.37, n.s.; SDS-score approx. 0) and emotional well-being (Z = -0.56, n.s.; SDS-score approx. 0). The mean SDS-score across all SF-36 subscales was 0.55 (SD = 0.74) indicating slightly better HRQoL in the first stroke VFD-sample compared to stroke patients 1 month post lesion.

The SF-36 scores of stroke patients 6 months post lesion were comparable to those of stroke patients with VFD only for the subscale vitality (Z = -0.2, n.s.; SDS-score approx. 0). In our sample, 5 of 8 SF-36 subscales (role limitations due to physical problems, general health perceptions, social functioning, role limitations due to emotional problems and emotional well-being) were significantly lower than in stroke patients with 6 months lesion age (Z-range -1.34 to -3.75, all *P *< 0.05; SDS<0). However, two subscales were still slightly better (physical functioning and bodily pain, Z = 1.95 and 4.57, *P *< 0.05; SDS>0). The mean SDS-score comparing both samples was -0.20 (SD = 0.84) indicating on average slightly worse HRQoL in VFD-patients compared to stroke patients 6 months post lesion (Figure 3).

**Figure 4:**
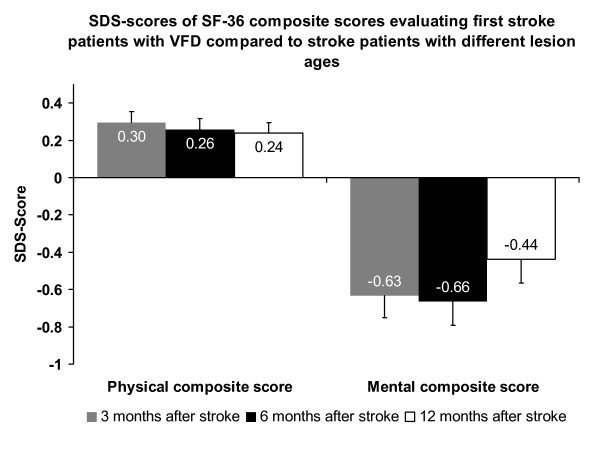
**SDS-scores for SF-36 composite scores of first stroke VFD-patients compared to stroke patients with different lesion ages**. Data of stroke patients with different lesion ages [35]. SDS was calculated as average SF-36 composite score in the first stroke VFD-sample minus average value of stroke patients three, six or twelve months post lesion divided by the standard deviation of the stroke groups with different lesion ages.

Results of SF-36 composite scores of VFD-stroke patients were also compared to results of stroke patients with different lesion ages (3, 6 and 12 months) (Figure 4). This reference data was originally published by Suenkeler et al. [35]. First-stroke patients with VFD showed better results for the physical composite score than stroke patients with different lesion ages (3 months: Z = -4.58, *P *< 0.0001; 6 months: Z = -4.21, *P *< 0.0001; 12 months: Z = -3.99, *P *< 0.0001). In contrast, SDS-scores indicated worse results for the mental composite score in VFD-patients compared to patients with different lesion ages (3 months: Z = -3.88, *P *< 0.0001; 6 months: Z = -3.77, *P *< 0.0001; 12 months: Z = -2.13, *P *< 0.05).

### Correlation analysis for QoL estimates with demographic and lesion characteristics

NEI-VFQ and SF-36 subscales were partially correlated with demographic variables, visual acuity and VFD-type (Table 2). No significant correlations with NEI-VFQ results were observed with demographic variables age, sex, lesion age and etiology. The VFD-type showed some low correlations (*P *< 0.1) with 4 of 12 NEI-VFQ subscales. The NEI-VFQ composite score and each subscale except ocular pain, driving and peripheral vision correlated significantly with visual acuity (r-range 0.27-0.48).

**Table 2:** Partial correlation coefficients between NEI-VFQ and SF-36 results of first stroke VFD patients with demographic and lesion variables, type of VFD and visual acuity

	R	Age	Sex	Lesion age	Etiology^1^	Visual field defect^2^	Visual acuity
NEI-VFQ (N)							

NEI-VFQ composite score (177)	0.51	0.01	-0.18	0.01	0.003	0.09	0.37 †
1. General health (173)	0.27	-0.09	-0.03	-0.01	0.14	0.24 §	0.27*
2. General vision (173)	0.52	-0.21	-0.13	-0.02	0.18	0.11	0.36 †
3. Ocular pain (175)	/	/	/	/	/	/	/
4. Near vision (177)	0.48	-0.01	-0.16	0.05	0.06	0.11	0.48 ‡
5. Distance vision (177)	0.35	0.05	-0.15	0.08	-0.05	0.12	0.35 †
6. Social functioning (177)	0.45	-0.004	-0.12	-0.003	-0.08	0.07	0.45 ‡
7. Mental health (174)	0.38	0.05	0.03	0.004	-0.02	-0.24 §	0.38 †
8. Role difficulties (175)	0.46	0.06	-0.2	0.1	0.03	0.15	0.29 *
9. Dependency (173)	0.37	-0.02	-0.19	0.04	-0.05	-0.24 §	0.37 †
10. Driving (153)	/	/	/	/	/	/	/
11. Color vision (172)	0.39	-0.2	-0.07	0.06	0.03	-0.22 §	0.39 †
12. Peripheral vision (175)	/	/	/	/	/	/	/

SF-36 (N)							

SF-36 physical composite score (169)	0.36	-0.2	-0.27 *	0.1	0.19	-0.05	0.16
1. Physical functioning (173)	0.47	-0.17	-0.28 *	0.02	0.16	-0.08	0.22 §
2. Role limitations (physical) (174)	0.42	-0.1	-0.15	0.21 §	0.19	-0.14	0.1
3. Bodily pain (174)	/	/	/	/	/	/	/
4. General health perceptions (173)	/	/	/	/	/	/	/
SF-36 mental composite score (169)	0.48	-0.04	0.37 †	0.01	0.004	-0.36 †	0.18
5. Vitality (176)	0.29	-0.1	0.20 §	0.18	0.03	-0.29 *	0.19
6. Social functioning (176)	0.32	0.02	0.24 §	0.003	-0.17	-0.32 †	0.12
7. Role limitations (Emotional) (170)	0.33	-0.04	0.22 §	-0.02	0.03	-0.33 †	0.15
8. Emotional well-being (176)	0.42	-0.04	0.16	-0.01	0.01	-0.26 *	0.31 *

* *P *< 0.05; † *P *< 0.01; ‡ *P *< 0.001; §*P *< 0.1. NEI-VFQ and SF-36 scores were partially correlated with demographic and lesion variables, type of VFD and visual acuity.

^1 ^The etiology was either ischemic (139) or hemorrhagic (38).

^2 ^The type of VFD was complete hemianopia (34), incomplete hemianopia (72), quadrantanopia (31), tunnel vision (5), scotoma (3), diffuse loss of vision (23) and visual field defect affecting three quadrants (9).

The mean eccentricity of detected stimuli in campimetry (i.e. of the intact visual field), which was analyzed in a subsample of patients with available digital visual field data, correlated significantly only with the peripheral vision NEI-VFQ scale (r = 0.26, p < 0.05, n = 90).

Emotional well-being was the only SF-36 scale which significantly correlated with visual acuity (r = 0.31; *P *< 0.05). SF-36 subscale physical functioning as well as the physical composite score and mental composite score were significantly correlated to the variable sex (r-range -0.27 to 0.37), but SF-36 subscales did not correlate with age, lesion age and etiology. Significant negative correlations were observed between the type of VFD and all 4 SF-36 subscales which compose the mental composite score. Therefore mental composite scores were descriptively compared between patients with different VFD-types tunnel vision patients (who typically suffer from the most extensive loss of visual field) expectedly had the lowest score of 39.45 compared to patients of all other VFD types (complete hemianopia 48.91; incomplete hemianopia 49.06; quadrantanopia 46.74; scotoma 46.12; diffuse loss of vision 43.59; visual field loss affecting three quadrants 45.66).

### Variance analyses of QoL estimates with the factor visual field size

**Figure 5:**
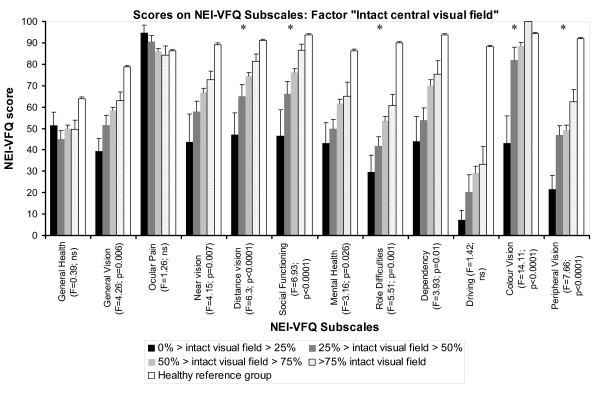
**Distribution of mean NEI-VFQ scores of first stroke VFD-patients according to the extent of intact central visual field**. The stroke sample was divided in four groups corresponding to the remaining intact central visual field size measured as the number of correctly detected stimuli in campimetry. The figure shows the distribution of mean NEI-VFQ scores of these four groups as well as results of healthy control persons [29]. A significant group difference was also observed for the NEI-VFQ composite score (see text).

The factor intact central visual field influenced every NEI-VFQ subscale except general health, ocular pain and driving (F-Range 3.16-14.11; all p < 0.05). Significant group effects below the adjusted significance level (0.00417) were observed for five NEI-VFQ subscales (Figure 5). A significant group difference was also observed for the NEI-VFQ composite score: 0-25% intact visual field size: 41.67 ± 19.43; 26-50%: 57.59 ± 19.58; 51-75%: 65.31 ± 15.42; 76-100%: 71.82 ± 12.45; (F = 7.66; p < 0.0001). In case of significant post hoc analyses, these revealed better NEI-VFQ results in patients with larger intact central visual field. Patients with more than 75% correctly detected stimuli in campimetry rated their VRQoL more than 30 points better (range 30.15-57.14; all p < 0.00417) than patients with an intact central visual field of 0-25% regarding the subscales distance vision, social functioning, role difficulties, color vision and peripheral vision as well as the composite score. Patients with an intact central visual field of 51-75% rated their VRQoL more than 20 points better than patients with an intact visual field of 0-25% in the subscales distance vision, social functioning, color vision and in the composite score (range 23.64-45.57; all p < 0.00417). Compared to patients with an intact visual field of 0-25%, patients with 26-50% estimated their VRQoL more than 40 points better for subscale color vision (39.14; p < 0.00417).

**Figure 6:**
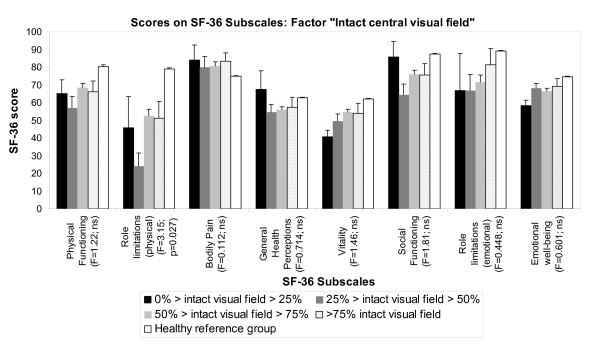
**Distribution of mean SF-36 scores of first stroke VFD-patients according to the extent of intact central visual field**. The stroke sample was divided in four groups corresponding to the remaining intact central visual field size measured as the number of correctly detected stimuli in campimetry. The figure shows the distribution of mean SF-36 scores of these four groups as well as results of healthy control persons [30]. There were also no significant group differences for SF-36 composite scores (see text).

Figure 6 shows SF-36 subscale scores corresponding to the factor visual field size. The intact central visual field affected only SF-36 subscale role limitations (physical) (F = 3.15; p < 0.05), but not significant at the adjusted significance level (0.00625). However, there were no significant *post-hoc *differences for this subscale. Further there were no significant group differences for SF-36 composite scores: physical composite score: 0-25% intact visual field size: 44.98 ± 10.08; 26-50%: 39.11 ± 11.92; 51-75%: 44.43 ± 9.91; 76-100%: 43.56 ± 8.76; (F = 1.89; p = 0.133) and mental composite score: 0-25% intact visual field size: 44.18 ± 9.79; 26-50%: 47.63 ± 10.35; 51-75%: 47.29 ± 11.94; 76-100%: 49.65 ± 12.22; (F = 0.376; p = 0.770).

### Variance analyses of QoL estimates with the factor visual acuity

**Figure 7:**
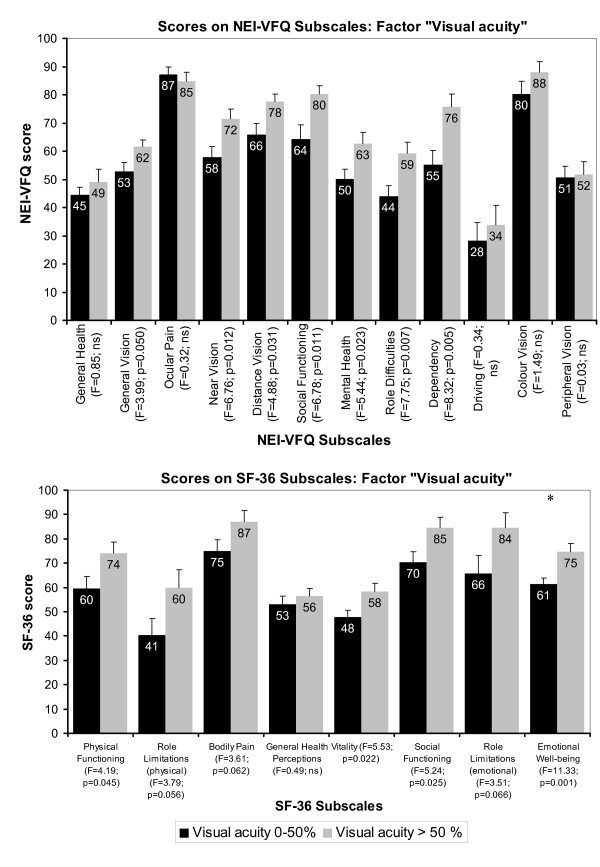
**NEI-VFQ and SF-36 score differences between visual acuity groups, when the first stroke sample was divided in two groups with visual acuity of 0-50% or >50%**. Visual acuity scores were percentage transformed. 0% corresponds to 0.4 decimal acuity respectively 0.4 LogMAR acuity.

Figure 7 shows NEI-VFQ and SF-36 subscale scores corresponding to the factor visual acuity. Stroke patients with VFD were assigned to one of two groups with either 0-50% or > 50% visual acuity. There was a trend for significant differences between both groups in all NEI-VFQ subscales except general health, ocular pain, driving, color vision and peripheral vision (F-range 3.99-8.32; all p < 0.05, but above 0.00417). Visual acuity influenced SF-36 subscales physical functioning, vitality, social functioning and emotional well-being (F-range 4.19-11.33; all p < 0.05, but only emotional well being below 0.00625) as well. In patients with better visual acuity higher NEI-VFQ and SF-36 results for the mentioned scales were observed. NEI-VFQ composite score significantly differed between both groups: 0-50%: 58.31 ± 19.64; >50%: 68.14 ± 12.62; (F = 5.67; p = 0.02), while the descriptive group difference for the SF-36 composite scores was lower: physical composite score: 0-50%: 40.90 ± 11.41; >50%: 45.24 ± 9.49 (F = 2.77; ns) and mental composite score: 0-50%: 45.36 ± 10.28; >50%: 51.69 ± 11.63 (F = 5.58; p = 0.02).

## Discussion

### Comparison of quality of life estimates between healthy controls and patients with VFD

The results of this study indicate a strong difference between VFD-patients and healthy controls which documents the substantial impact of vision impairment especially on subjectively perceived VRQoL. First, the observed SDS-scores were lower for VRQoL than for HRQoL (Figure 1) and second, VFD-patients differed from healthy controls in all dimensions of the NEI-VFQ except ocular pain (Table 1).

VFD-patients also showed significantly worse outcomes in all SF-36 dimensions than healthy controls except for the subscale role limitations due to emotional problems (Table 1 and Figure 2). Thus, general HRQoL as assessed with the SF-36 is still diminished 2.5 years after first posterior-parietal stroke that caused persisting VFD. The presented results complement those of previous studies [1-3]. However, these studies did not control for different etiologies of the VFD [2,3] or studied only a small sample [1,2].

### Comparison of quality of life estimates between general stroke samples and patients with VFD

Due to the availability of published HRQoL-results of a general stroke population [17] that naturally also included versatile and non-VFD functional impairments it was possible to compare stroke patients after different lesion ages and to concurrently rank subjectively perceived HRQoL of the investigated VFD-sample with this stroke sample which was investigated one and again six months after the lesion. One month post stroke patients experienced the lowest HRQoL, but their SF-36 scores improved by six months. Visually impaired stroke patients finally showed worse HRQoL than stroke patients six months post lesion, but better results than patients one month after stroke (Figure 3). This finding stresses the additional impact of VFD above stroke on diminished HRQoL. In future work a comparison of VRQol and HRQol between a stroke sample with VFD and one without should be attempted.

SF-36 results of VFD-stroke patients were also compared to results of stroke patients with different lesion ages (3, 6 and 12 months) of a second general stroke sample [35] (Figure 4). This comparison revealed better results of VFD-patients for the physical composite score but worse results for the mental composite score than for stroke patients in general at any point of investigation. This implies that first stroke patients with VFD after posterior-parietal strokes suffer from relatively isolated loss of vision that are less often accompanied by further neurological deficits such as motor impairment. In contrast, motor impairment is more often in a general stroke sample as published by Suenkeler et al. [35] resulting in worse physical composite scores than in the investigated sample of VFD-patients.

While quality of life estimates (both health-related and vision-related) were severely reduced in the investigated first stroke sample, Jobke et al. reported that no change in personality features could be observed in patients with visual field loss in general [36].

### Post stroke deficits, spontaneous recovery and etiology as possible confounders

A non-selected stroke population has a variety of post stroke deficits that can contribute to diminished HRQoL. It is well known that stroke patients recover rather well in non-visual functional domains such as speech and motor problems. Duncan et al. reported that most of the spontaneous motor recovery is completed within the first month after stroke [37]. Several studies allocate the greatest recovery from severe aphasia three months after stroke [38,39]. Spontaneous recovery of VFD often is only partial and also occurs within the first weeks up to six months after the damage, however only in about 38.4% of patients with homonymous hemianopia some spontaneous recovery was observed [40]. It has to be noted that the term "spontaneous recovery" is ambiguous because improvements that occur without intervention are usually non-differentiable from progress caused be early rehabilitation.

Rønning and Stavem reported that about 7-10% of their study participants had VFD post stroke [17]. The ratio of persisting VFD in their sample can only be estimated on the basis of published spontaneous recovery rates. There is a probability of partial spontaneous VFD recovery of 50% at one month post injury [40]. This improvement probability decreases to about 20% at six months post lesion [40]. Consequently, less than a decile of the general stroke population had (non-recovered) VFD six months post lesion.

An inclusion criterion of the VFD-sample was persisting visual field impairment and lesion ages beyond six months. The mean lesion age was 2.5 years, i.e. four times higher than the lesion age of the general stroke sample (6 months). Despite higher lesion age VFD-patients suffered from lower HRQoL than the general stroke sample 6 months post lesion. This demonstrates the significant contribution of VFD on reduced HRQoL that goes beyond the general stroke-related subjective impairments in HRQoL.

Another reason for this result may be that within the first weeks and even months after stroke patients become aware of constraints due to stroke, e.g. functional impairments, disabilities in working or everyday activities etc. A psychological hypothesis of post-stroke depression suggests that social and psychological stressors associated with stroke are the primary causes of depression [41]. Stroke patients can habituate to these stressors and develop compensatory strategies in the next few months implying that there is an additional component of self-perceived HRQoL increase during the period of spontaneous recovery and therapy-induced rehabilitation.

The factor etiology (ischemic vs. hemorrhagic infarction) does not seem to be related to self-reported visual impairment (Table 2) since no influence of etiology on VRQoL and HRQoL was observed in this and in a previous study [3].

### The influence of visual field loss and visual acuity on quality of life estimates

#### VRQoL

The results of the present study stress the impact of intact visual field extensions on QoL estimates. Patients with a larger intact central visual field showed higher scores on NEI-VFQ subscales which implies better subjectively perceived VRQoL (Figure 5). Further, the eccentricity of the VFD was reflected in subjective VRQoL. Better peripheral vision NEI-VFQ scores were observed in case of rather intact visual field in the periphery. This also demonstrated the content validity of the NEI-VFQ questionnaire. However, the correlation was only weak. This may be due to the fact, that the analysis was based on campimetry results that tested only positions within 30° of visual field. Higher correlations are likely to be observed when digital fullfield perimetry are obtained which were not available in the present study.

The present results are in accordance with previous studies [1,3] that reported an association of larger VFDs with worse self-evaluated visual functioning. Moreover, Papageorgiou also observed increasing VRQoL with advancing size of the area of sparing within the affected hemifield [1].

Our study also revealed that VRQoL was associated with visual acuity. Patients with better visual acuity also showed better VRQoL estimates as revealed by analyses of variance (Figure 7) and correlation analyses (Table 2). This relation between VRQoL as measured with the NEI-VFQ and visual acuity is in agreement with our previous study [3] where it was shown that visual acuity was related to VRQoL in all NEI-VFQ scales except general health, ocular pain and peripheral vision, but not to HRQoL (SF-36) when age was considered as a confounding variable.

#### HRQoL

While there was a distinct influence of visual field extensions on VRQoL, this was not the case for general HRQoL (Figure 6). Mental scores of HRQoL were particularly correlated with the type of the VFD (Table 2). A small, confined scotoma may have less negative impact on both overall visual function and cause less psychological distress than a quadrantanopia, hemianopia or diffuse loss since the type defines the size of the VFD as well. Patients with tunnel vision who typically suffer from the most extensive loss of visual field showed the worst scores for mental health subscales. However, this finding is based on only five subjects with tunnel vision. Similar results suggesting correlations between the size of VFD and NEI-VFQ subscales are reported for patients with postchiasmatic lesions [3] or glaucoma-patients [5]. Larger VFD cause larger restrictions in daily life and, besides subjective inconvenience, they could explain the decline on "mental" SF-36 subscales such as social functioning, emotional well-being and role limitations due to emotional problems (Table 2).

Concerning visual acuity, better ratings of emotional well-being were observed in patients with better visual acuity. Since emotional well-being also belongs to the mental component of the SF-36 this is additional evidence that both the size of the VFD as well as visual acuity predominantly influence mental rather than physical aspects of quality of life in general.

### Limitations of the study

The results of the present study have to be interpreted with some caution because co-morbidity as a possible confounding factor for QoL decline was not sufficiently controlled. As recently shown, co-morbidity in elderly visually impaired patients is associated with significant QoL decline [42]. In a large population-based epidemiological study different chronic conditions that often occur in parallel in the elderly were rank ordered across dimensions of HRQoL [43]. In this study visual impairments resulting from non-neurologic conditions were among diseases resulting in less adverse sequelae compared to cerebrovascular and neurologic conditions [43].

A further limitation of the present study is that the healthy reference samples [29,30] were not identical and this may be a source of variance. In future studies comparisons should be made to healthy control subjects recruited from a single population representative sample that answered both the NEI-VFQ and the SF-36.

An issue for further research is that the extent of the visual field defect as investigated in the presented study does not constitute the only factor influencing VRQoL. The eccentricity of the visual field loss may also play a major role since more central or even foveal visual field defects may result in worse VRQoL than peripheral defects of the visual field.

## Conclusions

VFD-patients after first posterior parietal-stroke showed severely reduced VRQoL and HRQoL even 2.5 years after the lesion. Compared with the investigated stroke patients with VFD the impairment level of stroke patients in general was larger concerning HRQoL 1 month post lesion but smaller at 6 months post lesion. This indicates that the stroke-related impairment level is significantly exacerbated by VFD. Since VFD occur in approximately 10% of stroke patients, patients with persisting VFD should be offered additional neuropsychological rehabilitation that may improve visual functioning [31] or supportive psychotherapeutic interventions because of their significantly reduced subjective mental health.

## Competing interests

The authors declare that they have no competing interests.

## Authors' contributions

CG developed the design of the study, carried out the organization of QoL and visual field data assessment of the stroke patients sample, performed the statistical analyses, and drafted and revised the manuscript. GHF conceived of the study, and participated in the design of the study. GHF also carried out the QoL data assessment of the healthy control sample and statistical analyses. BAS conceived of the study, and participated in its design. All authors read and approved the final manuscript.

## Supplementary Material

Additional file 1 **Overview of studies examining the relation between QoL measures and visual field loss after different etiologies**. The table presents study results for (I) visual field loss in glaucoma, (II) visual field loss after retinal lesions, (III) visual field loss after pre- and postchiasmatic lesions, and finally (IV) results of a population-based cross-sectional study. Information on the studied samples, and the way how visual fields and QoL were assessed are given. Note that statistical analyses and study results do not represent the whole content of the cited studies and are restricted on research questions targeting the relation between QoL and visual field loss. Click here for file
