# Supplementary material for: Vision-related quality of life in first stroke patients with homonymous visual field defects
Source: Health Qual Life Outcomes. 2010 Mar 26;8:33. doi: 10.1186/1477-7525-8-33 (PMC2859371; doi:10.1186/1477-7525-8-33)
Supplement: Additional file 1 — Overview of studies examining the relation between QoL measures and visual field loss after different etiologies. The table presents study results for (I) visual field loss in glaucoma, (II) visual field loss after retinal lesions, (III) visual field loss after pre- and postchiasmatic lesions, and finally (IV) results of a population-based cross-sectional study. Information on the studied samples, and the way how visual fields and QoL were assessed are given. Note that statistical analyses and study results do not represent the whole content of the cited studies and are restricted on research questions targeting the relation between QoL and visual field loss. [file 1477-7525-8-33-S1.DOC]

Additional file 1: Overview of studies examining the relation between QoL measures and visual field loss after different etiologies

| **Study** | **Sample (n)**  **Visual field examination**  **QoL measures** | **Statistical analyses and results** |
| --- | --- | --- |
| **I Visual field loss in glaucoma** | |  |
| Gutierezz et al. [5] | Glaucoma (147), Reference Group (44)  Humphrey Field Analyzer 24-2  NEI-VFQ, VF-14 & SF-36 | Spearman rank correlation coefficient:  Patients with glaucoma had significantly worse mean scores in 7 out of 11 NEI-VFQ-subscales  Correlations between the size of glaucomatous visual field loss for the better eye and NEI-VFQ in 7 of 11 subscales (r between -0.2 and -0.6) |
| Parrish et al. [6] | Glaucoma (147)  Humphrey Automated Perimeter  VF-14 & NEI-VFQ | Correlation between visual field loss and VF-14 scores r=-0.58)  Moderate correlations between visual field loss and NEI-VFQ (r between -0.56 and -0.6 in 3 subscales), modest correlations in 7 subscales (r between -0.32 and -0.55), weakly or no correlations in 3 subscales |
| Carta et al. [7] | Glaucoma (33); age-related macular degeneration / retinal vein occlusion (33) Cataract (34) Reference Group with minor refractive errors but no ocular disease (20)  Near and distance visual acuity with ETDRS chart; Visual field with Octopus 07-2LT program; Contrast sensitivity with Pelli-Robson chart  17-item Vision Function Questionnaire | Significant differences for total average score compared for cataract with glaucoma, reference group with macular degeneration/vein occlusion, glaucoma with reference group and macular degeneration with macular degeneration/vein occlusion;  Spearman rank correlation coefficient, multiple regression analyis:  Significant association of average score with distance visual acuity (r=0.489), near visual acuity (r=0.551), contrast sensitivity (r=0.550) and visual field size (r=0.384) |
| Jampel et al. [8] | Glaucoma (191), reference Group (46)  Humphrey Field Analyzer (Esterman binocular visual field test)  NEI-VFQ-25 | Multiple linear regression model, partial correlation coefficients:  Correlation visual field test and NEI-VFQ composite score (r = 0.32, p = 0.001)  Highest correlation with subscale social functioning (r = 0.38, p = 0.003) |
| Nelson et al. [9] | Glaucoma (47), reference group (19)  Humphrey Visual Field Analyzer, 24-2 threshold test,  Esterman binocular visual field test  Glaucoma Quality of Life – 15 (GQL-15) | Pearson correlation coefficient:  Significant correlation with perimetric mean deviation (r = -0.60, p < 0.0001) and visual field test (r = -0.39, p < 0.001) |
| Ringsdorf et al. [10] | Glaucoma (345, African American: 214, Whites: 131)  Advanced Glaucoma Intervention Study Scoring System (AGIS)  NEI-VFQ-25 | Spearman rank correlation coefficient:  Worse glaucoma-scores correlated with lower scores in 5 or 6 subscales (dependent on race and eye), r = -0.14 – -0.35 |
| McKean-Cowdin et al. [11] | Glaucoma (213), reference Group (2821)  Humphrey Automated Field Analyzer, Standard 24-2 program  NEI-VFQ-25 | Linear regression and analyses of covariance:  Correlations between visual field loss and VFQ composite scores and most subscales  Lower scores in patients with central visual field loss compared to peripheral loss of the visual field |

**Additional file 1: Overview of studies examining the relation between QoL measures and visual field loss after different etiologies, continued**

| **II Visual field loss after retinal lesions** | |  |
| --- | --- | --- |
| Szlyk et al. [12] | Central Juveline macular dystrophies (72); peripheral retinitis pigmentosa (120)  Goldmann perimeter with II-4-e target  33-item self report questionnaire | Spearman rank correlation coefficient:  Correlation with visual field loss for 20 of 33 questions (r between -0.20 and -0.52) for juveline macular dystrophies  Chi2 analysis: differences between groups in 25 items (p = 0.001 – 0.01) |
| Deramo et al. [13] | Central retinal vein occlusion (CVO) (51)  Diabetic retinopathy  Low vision from a variety of causes  Reference Group without ocular desease  Snellen visual acuity  NEI-VFQ-25 | Kendall tau-b correlation coefficient, Spearman rank corellation coefficient:  Lower scores in CVO than in a control group (except ocular pain) but similar to those with diabetic retinopathy,  Strong correlations of NEI-VFQ results with visual acuity for the better eye (9 subscales),  CVO patients with a visual acuity of 20/25 or better in the unaffected eye had significantly lower scores in 8 subscales compared to reference group |
| **III Visual field loss after pre- and postchiasmatic lesions** | | |
| Noble et al. [14] | Optic neuropathy in patients with multiple sclerosis (34)  Humphrey Automated Field Analyzer, 30-2 program  NEI-VFQ-25 | Spearman rank correlation coefficient:  correlation of NEI-VFQ composite score and visual field size( r = 0.53, p = 0.003) |
| Papageorgiou et al. [1] | Hemianopia (33)  Reference group (360)  OCTOPUS 101 perimeter, Binocular semi-automated kinetic perimetry (SKP)  NEI-VFQ-25 | Spearman rank correlation coefficient:  Lower scores in 7 out of 12 subscales and the composite score,  Moderate to weak correlations between area of sparing and composite score (r=0.38) respectively 6 of 12 subscales (r = 0.25 – 0.51) |
| Gall et al. [2] | Patients with postchiasmatic visual field loss (24)  Rodenstock Perimat 206  NEI-VFQ-39 & SF-36 | Spearman rank correlation coefficient:  Correlation with visual field size in 10 out of 12 subscales (r = 0.44 – 0.68) and the composite score (r = 0.67) |
| Gall et al. [3] | Patients with postchiasmatic visual field loss (312)  Humphrey Automated Field Analyzer, Twinfield Oculus, Rodenstock Perimat 206  NEI-VFQ-39 & SF-36 | Multiple linear regression model, partial correlation coefficients:  Correlation visual field test and NEI-VFQ composite score (r = 0.32, p = 0.001)  Highest correlation with subscale social functioning (r = 0.38, p = 0.003) |
| **IV Visual field loss in a population-based cross-sectional study** | | |
| McKean-Cowdin et al. [4] | Subjects without visual field loss (2886) compared to patients with mild (1910) and moderate to severe visual field loss  Humphrey Automated Field Analyzer II, Standard 24-2 program  NEI-VFQ-25 | Linear Regression ß-coefficients:  4 to 5 dB differences in visual field loss were associated with five-point differences in VFQ, linear relationship between severity of visual impairment and NEI-VFQ subscale and composite scores |
